# Exploring the Impact of TREM2 in Tumor-Associated Macrophages

**DOI:** 10.3390/vaccines10060943

**Published:** 2022-06-14

**Authors:** Darya Khantakova, Simone Brioschi, Martina Molgora

**Affiliations:** Department of Pathology and Immunology, Washington University School of Medicine, St. Louis, MO 63110, USA; d.khantakova@wustl.edu (D.K.); s.brioschi@wustl.edu (S.B.)

**Keywords:** TREM2, tumor-associated macrophages, cancer, immunotherapy

## Abstract

**Simple Summary:**

TREM2^+^ macrophages were recently reported to be highly enriched and associated with immunosuppression in various cancer types. Hence, TREM2 targeting represents a new promising approach for cancer treatment that is based on reprogramming of tumor-associated macrophages to reshape anti-tumor immunity and overcome resistance to current therapies.

**Abstract:**

Tumor-associated macrophages (TAMs) represent a key component of the tumor microenvironment and are generally associated with immunosuppression and poor prognosis. TREM2 is a transmembrane receptor of the immunoglobulin superfamily expressed in myeloid cells. TREM2 has been extensively studied in microglia and neurodegenerative diseases and recently emerged as a marker of pro-tumorigenic macrophages. The accumulation of TREM2-expressing TAMs was reported across numerous cancer patients and tumor models. TREM2 genetic blockade or TREM2 targeting with antibodies resulted in improved tumor control, enhanced response to anti-PD1, and significant changes in the tumor immune landscape. Preclinical studies paved the way for an ongoing clinical trial with a TREM2 depleting antibody and inspired further exploration of TREM2 targeting therapies. Here, we review the current knowledge about the impact of TREM2 in cancer, with an emphasis on the TREM2^+^ macrophage signature across different cancer types, the contribution of TREM2 to TAM phenotype and function, and the promising effects of TREM2 modulation.

## 1. Introduction

TREM2 (Triggering Receptor Expressed on Myeloid cells 2) is a type I transmembrane receptor expressed on myeloid cells, primarily macrophages. The extracellular region of TREM2 contains an immunoglobulin-like domain for ligand binding and a stalk region with a cleavage site for the metalloproteases ADAM10 and ADAM17 [1]. TREM2 has a short intracellular tail lacking a signaling domain. Hence, TREM2 requires the adaptor proteins DAP12 and DAP10 for intracellular signaling [2]. DAP12, encoded by *TYROBP*, induces the recruitment and activation of the spleen tyrosine kinase (SYK), which in turn promotes a signaling cascade including PLCG2, PI3K, and AKT, resulting in the elevation of intracellular Ca^2+^ and mTOR activation [3,4,5]. DAP10 is homologous to DAP12, encoded by *HCST*, and contains a binding sequence for PI3K [6]; therefore, this branch of the TREM2 pathway may primarily rely on this kinase. Overall, TREM2-dependent signaling promotes protein synthesis and metabolic processes involved in immune activation and cell survival. Putative TREM2 ligands include anionic lipids, lapidated apolipoprotein (especially APOE), and membrane phospholipids such as phosphatidylserine exposed on apoptotic cells [7,8,9]. A soluble form of TREM2 (sTREM2) was identified and can be detected in serum and cerebrospinal fluid (CSF) [10]. TREM2 has been extensively studied in microglia because of its significant impact on neurodegenerative disorders, as well as physiological processes in the brain [5]. 

Recently, attention has been focused on TREM2-expressing tumor-associated macrophages (TAMs). TAMs represent a major component of the tumor microenvironment (TME), being primarily immunosuppressive and associated with poor prognosis [11,12,13]. TAMs can affect tumor growth, directly and indirectly, promoting tumor cell survival and proliferation, as well as angiogenesis, invasiveness, and immune evasion. The therapeutical targeting of TAMs to either reduce TAM frequency or modulate TAM phenotype and function is one of the biggest challenges for cancer immunotherapy [11,13]. TREM2 emerged as a specific marker of TAMs, consistent throughout different human cancers and tumor models, and strongly associated with immunosuppression. TREM2, therefore, represents a promising therapeutic target, holding great potential for immunotherapy treatments, possibly in combination with T-cell-targeting therapies. Here, we will provide an overview of the impact of TREM2 on myeloid cells and then focus on the most recent literature on TREM2 in tumor immunology. We will discuss the TREM2^+^ macrophage signature in human cancers and the contribution of TREM2 to TAM phenotype and function. We will also highlight the impact of TREM2 targeting in tumor models and its clinical applications.

## 2. TREM2 in Myeloid Cells, Historical Perspective

TREM2 was initially cloned from human PBMC in 2000 [14,15], followed by the identification of the mouse orthologous soon after [16]. Early studies further revealed that the adaptor molecule DAP12 associates with TREM2 [15], and its expression is upregulated on monocyte-derived alternatively activated macrophages [17], thus suggesting that TREM2 might be involved in macrophage activation.

Null mutations in either *TREM2* or *TYROBP* genes were reported to cause a rare form of early-onset neurodegenerative disorder known as polycystic lipomembranous osteodysplasia with sclerosing leukoencephalopathy (PLOSL), or Nasu–Hakola Disease (NHD) [18,19,20]. Affected patients often develop osteoporosis with bone cysts and fractures, frontotemporal atrophy, basal ganglia calcification, and white matter degeneration within the third-to-fourth decade of life [21,22]. The etiology of the disease remains unclear. Microglia are the only cells in the human brain expressing TREM2 or DAP12 [23], suggesting that microglial dysfunction might play a role in the onset of the disease. Furthermore, NHD patients exhibited deficient differentiation of osteoclasts *in vitro* [24,25], raising the possibility that juvenile osteodysplasia might be associated with neurodegeneration and dementia in adulthood. More recently, TREM2 received growing attention because of its genetic association with Alzheimer’s disease (AD). Multiple GWAS studies have consistently linked polymorphic variants of TREM2, especially the single nucleotide polymorphism (SNP) causing R46H substitution, to late-onset AD [26,27,28,29]. R47H polymorphism appears to reduce ligand binding and generates a hypofunctional variant of the wild-type TREM2 [8,30,31,32]. Currently, the mechanistic basis for the correlation between TREM2 defects and AD pathology is under investigation. 

**Figure 1:**
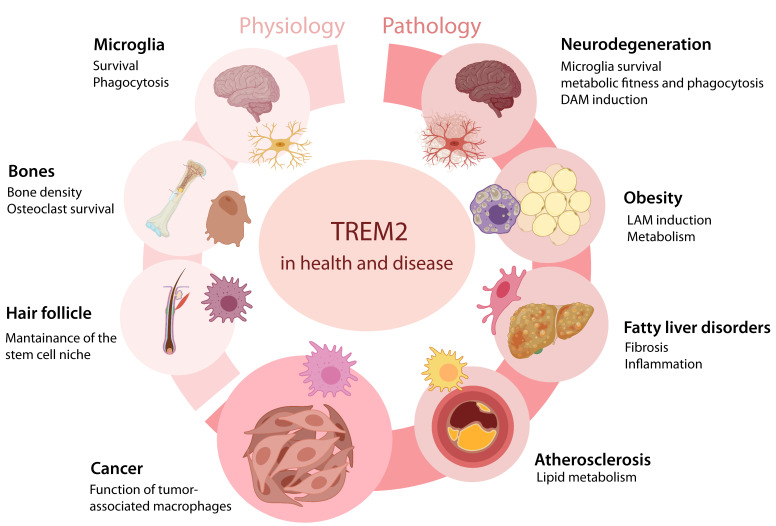
**Role of TREM2 in physiological and pathological conditions.** TREM2 is expressed in macrophages across different tissue conditions and contributes to several physiological and pathological processes. DAM, disease-associated microglia. LAM, lipid-associated macrophages.

At the functional level, TREM2 appeared to play different roles depending on the cell type and pathological settings [33,34,35,36,37,38,39,40,41,42] (Figure 1). Nevertheless, the induction of cell survival and metabolic programs often emerged as a conserved effector function in multiple contexts [2,5,8]. More recently, TREM2 also has emerged as a key player in lipid metabolism. Trem2-deficient mice exhibited accumulation of intracellular cholesterol in brain microglia during demyelination [36,43], along with defective myelin repair in the central nervous system (CNS) white matter [40,41,44,45]. These findings indicate that TREM2-induced lipid metabolism in microglia critically regulates myelin turnover. TREM2 is also known to orchestrate microglial reactivity during amyloid pathology. In mouse models of AD, wild-type microglia proliferate and migrate in the proximity of the amyloid plaques, thus forming a cellular barrier encasing the amyloid deposits [46]. Conversely, Trem2-deficient microglia fail to cluster around amyloid plaques, thus resulting in impaired barrier function and increased neuronal damage [8,35,47,48]. 

Furthermore, the absence of Trem2 impaired activation of the microglial transcriptional program in response to amyloid beta pathology and neurodegeneration [33,34]. Notwithstanding, it remains unclear whether such defects in microglia depend on accelerated cell death or insufficient response towards the amyloid lesions. 

In the last decade, the introduction of single-cell RNA sequencing (scRNAseq) technology revolutionized the field of medical sciences, and the opportunity to study the transcriptome of immune populations at the single-cell level in an unbiased manner pioneered the discovery of TREM2-expressing cells across different tissues and conditions. For instance, TREM2 is highly expressed in peripheral macrophage populations involved in physiological conditions, metabolism, host defense, and different pathologies (Figure 1). In microglia, TREM2 was reported to be enriched in the disease-associated microglia (DAM) subset, which accumulates during neurodegenerative diseases. In fact, *Trem2* expression in microglia was associated with the DAM signature (*Apoe*, *Cd9*, *Cd11c*, *Cd63*, *Clec7a*, *Lpl*, *Cst7*, *Tyrobp*, *Fth1*, *B2m*) and with the downregulation of steady state genes (*Cx3cr1*, *P2ry12*, *P2ry13*) [23,33]. In the adipose tissue, *Trem2* drives the accumulation of a population of lipid-associated macrophages (LAMs) that promotes phagocytosis, energy metabolism, and lipid catabolism, providing a protective response in the context of a high-fat diet and the loss of metabolic homeostasis [37]. LAMs were characterized by the enriched expression of *Trem2*, *Cd9*, *Lpl*, and *Cd36*, having a genetic signature that is reminiscent of DAM [37]. Trem2^+^ macrophages are also enriched in atherosclerotic plaques and specialize in lipid catabolism [49]. In liver disorders, a TREM2^+^CD9^+^ subset of monocyte-derived macrophages (also referred to as “scar-associated” macrophages) expands during liver cirrhosis and contributes to pathogenesis [50,51,52]. During acute pulmonary viral infection, the release of sTrem2 inhibits macrophage apoptosis and causes chronic inflammation [53]. In the skin, sTrem2 contributes to the inhibition of hair growth. Dermal Trem2^+^ macrophages were shown to release oncostatin M (OSM), which maintains hair follicle stem cell quiescence [54]. Moreover, Trem2 is expressed by osteoclasts and is involved in bone density and osteoclast survival, providing a potential mechanism for the bone lesions associated with NHD [55].

## 3. Contribution of TREM2 to the Immune Response in Tumors

### 3.1. TREM2^+^ Macrophages Are Enriched in Human Cancers and Associated with Immunosuppression

**Figure 2:**
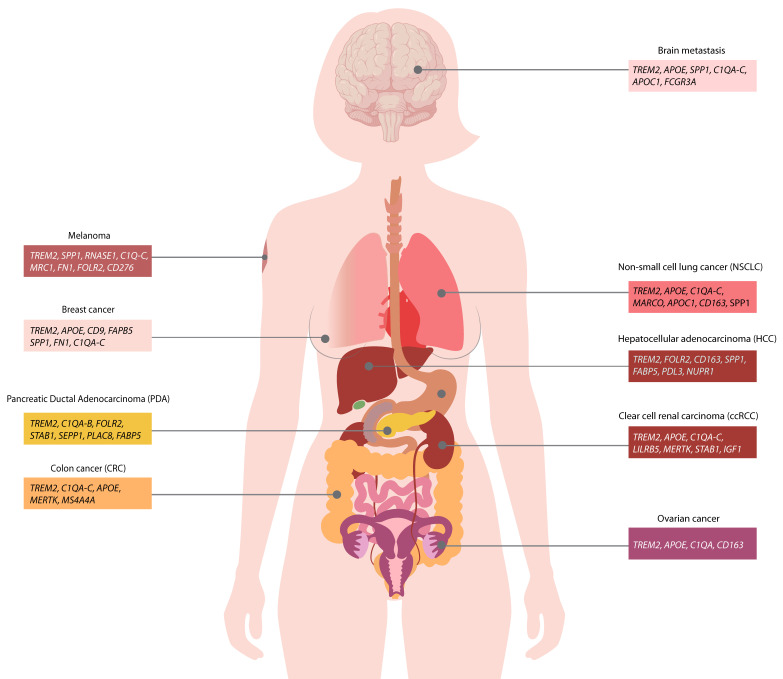
**TREM2-expressing macrophages are accumulated in different human tumors.** TREM2^+^ macrophage signature is shared across numerous human cancers and is associated with immunosuppression.

TREM2 was recently described as a marker of immunosuppressive TAMs, being part of a gene signature that was partially consistent across different tumor types and models (Figure 2). We recently characterized the TREM2 contribution to the TAMs phenotype using the MCA1956 sarcoma model in Trem2-deficient mice [38]. Trem2 expression was detected by scRNAseq across all macrophage clusters. *Cx3cr1^+^* macrophages expressed the highest levels of *Trem2* and were strongly reduced in tumors of Trem2 knockout mice. This cluster was enriched for genes associated with immunosuppression (*Mrc1*, *Mertk*, *Cd81*), complement genes (*C1qa*), chemokines (*Ccl2*, *Ccl7*), extracellular matrix remodeling (*Chil1*), and angiogenesis (*Vcam1*) [38].

#### 3.1.1. Lung Adenocarcinoma

One of the first pieces of evidence of TREM2 expression in TAMs was from Lavin et al., who performed an scRNAseq analysis of myeloid cells from 18 treatment-naïve patients with stage I lung adenocarcinoma lesions [56]. A simultaneous single-cell analysis of immune cells from tumor lesions, normal lung tissue, and the blood of each patient identified a subset of TREM2-expressing macrophages specifically enriched in tumors. Along with *TREM2*, these cells were enriched for genes involved in lipid metabolism (*APOE*), immunosuppression (*MARCO*, *CD163*, *CD81*), and the complement cascade (*C1QB*), suggesting that TREM2 specifically marks a subset of tumor-specific immunosuppressive macrophages [56]. ScRNAseq analysis of early-stage non-small cell lung cancer (NSCLC) showed that the TREM2 transcript was present in all the identified populations of monocyte-derived macrophages [57]. TREM2 was particularly enriched along with the immunosuppressive gene *LILRB4* [57,58]. Interestingly, in this context a subset of monocyte-derived macrophages was enriched for *SPP1* and, in fact, was part of the immune activation program that was associated with a better response to anti-PD-L1 [57]. 

TREM2^+^ macrophages were also identified in samples of advanced-stage NSCLC that were obtained from patients before and after initiating systemic targeted therapy [59]. ScRNAseq analysis revealed that TREM2^+^ TAMs were enriched for lipid metabolism genes (*APOE*, *APOC1*, *CTSD*, *PLD3*), complement components (*C1QA-C*), as well as *SPP1* and *FOLR2* genes. This population was slightly decreased upon treatment, but there was no difference in progressive disease versus residual/stable disease. This indicates that targeted therapy promotes TAM modulation, but in this specific context, namely type of treatment and advanced stage of disease, TREM2^+^ TAMs were not associated with a favorable response [59]. A different study analyzed a large dataset of NSCLC patients, and TREM2^+^ TAMs were shown to be enriched for *APOE*, *APOC1*, *SPP1*, *C1QB-C*, *APOC2*, and *MARCO* genes, as compared to TREM2^-^ TAMs [60]. Moreover, TREM2^+^ TAMs expressed higher levels of immunosuppressive markers such as CD206, ARG-1, and IL-10 by flow cytometry. CD8 T cells in tumors with high TREM2^+^ TAMs had a diminished production of effector molecules, such as Perforin (PRF1) and TNF-a. Using NSCLC tissue microarrays, it was noted that higher TREM2 expression positively correlated with advanced tumor stages, shortened overall survival, and recurrence-free survival [60]. Moreover, patients with progressive disease upon anti-PD-1 therapy showed a higher proportion of TREM2^+^ cells compared to patients with stable disease or partial response. This supports the potential prognostic value of TREM2 in NSCLC patients, as well as the relevance of TREM2 targeting in anti-PD-1 non-responders [60]. Overall, TREM2^+^ macrophages were shown to be accumulated in NSCLC tumors and associated with an immunosuppressive LAM-like signature, as well as poor patient outcome. 

#### 3.1.2. Breast Cancer

A deeper characterization of the immune landscape of breast cancer (BC) subtypes can help identify new targets and promote the development of precision medicine approaches [61]. Multiple scRNAseq studies provided an extensive overview of the immune composition in different subtypes of BC [61,62,63,64,65]. It was shown that TAMs constitute a major component of BC tumors, and TREM2 appeared to be highly expressed in TAM populations across different subtypes in numerous studies [61,62,63,64,65]. In treatment-naïve patients with diverse subtypes, TREM2-expressing macrophages accounted for 30–40% of total myeloid cells [61]. The TREM2 transcript was correlated with genes associated with lipid metabolism (*CD9*, *FABP5*, *APOE*), immunosuppression (*CD81*, *FN1*, *CD276*, *MARCO*, *SPP1*), and complement components (*C1QB*), as well as chemokine genes associated with immune stimulation (CCL3) [61,62]. ScRNAseq analysis of 12 treatment-naïve luminal BC patients on primary tumors, metastatic lymph nodes, and blood defined two phenotypically distinct macrophage populations based on the expression of FOLR2 and TREM2. TAMs were present in both tumor and metastatic lymph nodes and were defined based on the shared expression of *APOE*, *APOC1*, and *C1QA*-*C*, and the mutually exclusive expression of *TREM2/CADM1* and *FOLR2* [63]. In line with previous studies, TREM2 macrophages expressed *SPP1* and were associated with the LAM signature (*FABP5*, *CD9*, *MSR1*), as well as immunosuppressive genes (*FN1*) and type I IFN-induced genes (*ISG15*, *IFI27*, *IFI6*). FOLR2 macrophages were enriched in tumors with high CD8 T cell infiltration and associated with favorable clinical outcomes, suggesting that they might promote anti-tumor immunity in luminal BC. By flow cytometry, the ratio of FOLR2 to TREM2 macrophages was reduced during tumor progression in primary tumors and metastatic lymph nodes. Histological analysis showed that FOLR2^+^ macrophages were primarily localized in the tumor stroma, while TREM2^+^ TAMs were found both in the stroma and in tumor nests, especially along the invasive margin and forming cell clusters. This could suggest that TREM2^+^ macrophages might be engaged in closer bidirectional interactions with tumor cells, being therefore more exposed to tumor-derived immunosuppressive factors, and in turn affecting tumor cell survival, proliferation, and invasiveness. Moreover, hierarchical clustering suggested the transcriptional proximity of TREM2^+^ TAMs to CD14^+^CCR2^+^ monocytes in breast tumors, implying that they are monocyte-derived macrophages [63]. Altogether, TREM2 and FOLR2 might define two functionally distinct TAM populations, highlighting TAM heterogeneity *in vivo* and paving the way for TREM2 and FOLR2 targeting in BC patients. Further understanding of TAM diversity in other BC subtypes will be of great interest to efficiently modulate macrophage phenotypes and promote anti-tumor immunity.

To assess the changes related to treatment response in the tumor microenvironment, a scRNAseq study involved a cohort of 22 patients with advanced triple-negative breast cancer (TNBC), treated with chemotherapy alone or in combination with anti-PD-L1 [64]. Macrophage frequency was higher at baseline in non-responders in the combination therapy cohort. The analysis of differentially expressed genes (DEG) in macrophages of pretreatment samples from responders versus non-responders showed that *TREM2*, *SPP1*, *FN1*, *C3*, and *LDHA* genes were enriched in non-responders. This highlights the potential prognostic value of TREM2^+^ macrophages in TNBC patients. TREM2^+^ macrophages were positively correlated with regulatory T cell accumulation and negatively associated with cytotoxic CD8^+^ T cells [64]. In anti-PD-1-treated BC patients, TREM2 TAMs (*TREM2*, *CX3CR1*, *C3*, *PLD4*, *CCL3*) were inversely correlated with T cell clonal expansion, suggesting an association with poor response to anti-PD-1 therapy [65]. The enrichment of TREM2 macrophages was correlated with worse survival in luminal and triple-negative BC patients, as well as in a meta-analysis of different cohorts [38,61,63]. Altogether, TREM2 macrophages were associated with immunosuppression, LAM signature, poor T cell infiltration, worse outcome, and reduced response to ICT in BC. 

#### 3.1.3. Hepatocellular Carcinoma 

In hepatocellular carcinoma (HCC) patients, the contribution of TREM2 macrophages seems to be more controversial. In line with other studies, TREM2 TAMs were highly enriched in tumors but not in adjacent liver tissue and were an indicator of shorter survival in HCC patients [66], suggesting the immunosuppressive role of TREM2 in the liver TME. TREM2^+^ macrophages expressed *FOLR2*, *CD163*, and *C1Q-C*, and also genes associated with a LAM-like signature, such as *FABP5*, *PLD3*, *LGALS3*, *CD36*, and *SPP1* [66]. 

On the contrary, a different study reported the presence of fetal liver-like *FOLR2^+^ CD163^+^* TAMs, and *SPP1^+^ TREM2^+^* TAMs, with the FOLR2-enriched population being the one associated with more immunosuppressive interactions [67]. The TREM2 macrophage signature was enriched for *FABP5* and *NUPR1* and was reminiscent of the LAM-like signature (*APOC1*, *LGALS1*, *CSTB*, and *PLD3*). Collectively, this suggests that TREM2 macrophage impact in tumors can be influenced by tissue-specific factors. This highlights the importance of exploring TAM heterogeneity in different organs and tumor types to determine a specific phenotype and function of different TAM subsets. 

#### 3.1.4. Other Solid Tumors

TREM2^+^ macrophages have emerged in a variety of other solid cancers, highlighting the universal role of TREM2 as a marker of immunosuppressive TAMs. In colorectal cancer (CRC) patients, scRNAseq analysis of matched primary tumor and liver metastasis was performed to characterize the immune landscape diversity and elucidate factors contributing to metastasis [68]. Among monocyte/macrophage clusters, three were defined as immunosuppressive and named “malignancy-associated”: C1QC^+^ (*TREM2*, *C1QA-C*, *MERTK*, *MS4A4A*, *APOE*), SPP1^+^ (*FN1*, *VEGFA*, *IL1RN*), and MKI67^+^ (*HMGN2*, *H2AFZ*) [68,69]. TREM2 was expressed in “malignancy-associated” macrophages, with the highest levels in the C1QC^+^ subset that specifically accumulated in the tumor compared to the healthy counterpart. C1QC^+^ and SPP1^+^ macrophages both accumulated in CRC liver metastasis, with the latter being the most enriched in metastatic tissue compared to primary tumor [68]. SPP1+ macrophages showed the strongest angiogenesis score, and were associated with an unfavorable prognosis in colon adenocarcinoma, rectum adenocarcinoma, and CRC. C1QC+ TAMs were associated with phagocytosis and, notably, expressed the Th1 cytokines CXCL9 and CXCL10 [68]. In a TCGA CRC cohort, TREM2 expression was associated with worse overall survival and correlated with immunosuppressive genes (i.e., *MSR1*, *HAVCR2*) and lipid metabolism genes (*APOC1*, *APOE*, *OLR1*) [38]. To conclude, the C1QC+ (TREM2^high^) and SPP1+ macrophages possibly represent two phenotypically distinct TAM populations in CRC and CRC-derived liver metastasis, but their functional role still needs further investigation. 

A meta-analysis of an scRNAseq dataset from melanoma patients treated with checkpoint inhibitors (anti-PD-1, anti-CTLA4 + anti-PD-1 or anti-CTLA4) revealed that TREM2^high^ TAMs were 15.1-fold more abundant in non-responders [70]. The TREM2^high^ TAMs signature was associated with the enrichment of the *SPP1*, *RNASE1*, *MT1G*, *SEPP1*, *FOLR2*, *APOC2*, and *C1QA-C* transcripts, along with other genes associated with immunosuppression (*MRC1*, *FN1*, *CD276*, *MMP12*, *TGFB2*).

In clear cell renal carcinoma (ccRCC) patients, TREM2^+^ TAMs were defined by the expression of *TREM2*, *APOE*, and *C1QA-C*, along with other macrophage markers associated with immunosuppression, such as *LILRB5*, *MERTK*, *STAB1*, and *IGF1* [71]. A histological examination revealed that this population (defined as CD68/CD163+C1Q+TREM2^+^) was present only in tumor tissues and was significantly associated with post-surgical disease recurrence. 

A meta-analysis of an scRNA-seq dataset from pancreatic ductal adenocarcinoma (PDA) tumors showed that TREM2^+^ macrophages were enriched in PDA tumors compared to normal adjacent pancreas tissue and expressed *C1QA-B*, *FOLR2*, *STAB1*, and *SEPP* genes [72]. 

Multiple pan-cancer studies also identified the TREM2 signature. Mulder and colleagues performed an integrated analysis of scRNAseq data from 13 different tissues (healthy and diseased) across 41 datasets to define conserved gene signatures of human mononuclear phagocytes [73]. A comparison of tumors to healthy tissues showed an accumulation of TREM2^+^ macrophages in all the analyzed tumors (lung, colon, liver, breast, stomach, and pancreas datasets), and TREM2^+^ TAMs were also enriched at the patient level in tumor tissues (liver, lung, and colon datasets). In line with previous reports, TREM2^+^ macrophages showed an association with *SPP1* and lipid metabolism genes (*CD9*, *APOC1*, *CD63*, *FABP5*, *LGALS3*, *LIPA*), and similarities with the monocyte signature, suggesting their monocytic origin [73]. Another study in 10 different human tumors revealed five clusters of TAMs that expressed *TREM2* [74], with two of them (*PDGFB^+^* and *FOLR2^+^* TAMs) comprising up to 30% of the total monocyte/macrophage compartment, and being highly associated with exhausted CD8^+^ T cells [74]. Moreover, flow cytometry analysis of 49 different human tumors showed that TREM2 expression was enriched in TAMs compared to the other myeloid populations in the TME. Interestingly, ovarian cancer emerged as the tumor type having the highest TREM2 expression (above 1000 geometric mean fluorescent intensity), combined with TAM frequency (above 40% of the CD45^+^ infiltrating cells). Histological analysis showed that TREM2 expression strongly correlated with disease stage in human ovarian cancer. This highlights the importance of patient stratification to score TREM2/TAM enrichment and identify tumor types that may be more responsive to TREM2-targeting therapies [74]. 

Moreover, a recent study examined brain metastases from different cancer types (melanoma, breast, lung, ovarian, colorectal, and renal cancer) and revealed a cluster of TREM2^+^ macrophages (*APOE*, *SPP1*, *C1QA-C*, *APOC1*, *FCGR3A*) [75], supporting a possible impact of TREM2 in the metastatic process. Finally, an integrated transcriptomic analysis from multiple datasets of human cancers identified TREM2-expressing TAMs in lung, colon, liver, breast, stomach, pancreas, ovaries, skin, kidney, as well as head and neck cancers [73,74,76,77]. 

Collectively, although there are many factors affecting TAM transcriptional signature (i.e., tissue, disease stage, treatment, methodology), TREM2 clearly emerges as a core gene of immunosuppressive TAMs across numerous cancer types (Figure 2). Importantly, this subset of TAMs consistently exhibited transcriptional programs associated with lipid metabolism and immunosuppressive functions, and was often correlated with poor prognosis and reduced response to therapy. Future studies will be needed to further elucidate the functional relevance of TAM heterogeneity *in vivo* and help develop TAM-targeting treatments in different tumors.

### 3.2. TREM2 Modulates Tumor-Associated Macrophage Phenotype and Function

Our laboratory and Ido Amit’s laboratory independently described the role of TREM2 in tumor immunity using either TREM2 genetic ablation or pharmacological blockade [38,78]. TREM2 deficiency was associated with reduced tumor growth in several transplantable models: MCA-derived sarcoma (MCA1956 and MCA206 cell lines), MC38 colorectal carcinoma, and PyMT breast cancer [38,78]. Consistently, TREM2 expression in the tumor was correlated with reduced survival in patients with CRC and TNBC [38].

We showed that TREM2 was mainly expressed in TAMs, in sections from patients and in the MCA1956 murine model [38]. The absence of TREM2 caused significant changes in the tumor immune landscape, in both the myeloid and the lymphoid compartments. ScRNAseq analysis revealed that the presence of *Trem2* promoted the enrichment of immunosuppressive macrophage subsets (*Cx_3_cr1^+^* and *Mrc1^+^* macrophages), whereas TREM2-deficient mice had an accumulation of immunostimulatory macrophage myeloid cells (*Cd83^+^ Cxcl9^+^* macrophages) [38]. Furthermore, the absence of TREM2 increased the overall frequency of lymphoid cells, with a considerable accumulation of activated CD8 T cells. Accordingly, CD8, but not CD4 T cell depletion, nullified the beneficial effects of TREM2 deletion [38]. Katzenelenbogen et al. also reported TREM2 expression in myeloid cells with immunosuppressive activity in the MCA205 sarcoma model [78]. ScRNAseq analysis of the tumor infiltrate identified two subsets of macrophages with an immunoregulatory phenotype, annotated as TAMs and Mregs (regulatory myeloid cells), respectively. TAMs were enriched for *C1qa*, *Spp1*, *Cx3cr1*, and *Apo1*, while Mregs expressed high levels of *Gpnmb*, *Il7r*, *Hilpda*, *Vegfa*, *Hmox1*, and *Clec4d*. Both populations co-expressed *Trem2*, *Arg1*, *Cx3cr1*, *Mrca1*, and *Pdpn* [78]. Consistent with our findings, TREM2-deficient mice showed reduced tumor growth, along with decreased Mreg and increased TAM population frequencies [78]. Furthermore, *Trem2*-deletion increased the infiltration of NK cells and cytotoxic T cells in the tumor, whereas PD-1- and Tim-3-expressing T cells were reduced [78].

To test the impact of TREM2 pharmacological blockade in tumors, we developed a recombinant monoclonal antibody (178 mAb) that specifically recognizes murine TREM2 and carries a mutation in the Fc portion to prevent Fc effector functions [38]. Mice treated with the anti-TREM2 mAb displayed reduced tumor growth in the MCA sarcoma model and TME that partially resembled the TREM2 knockout condition [38]. Upon 178 mAb treatment, immunosuppressive *Mrc1^+^* macrophages were reduced, while *Nos2+* immunostimulatory macrophages were enriched, and T cells produced higher levels of IFNγ and TNFα [38]. TREM2 deletion and anti-TREM2 mAb treatment were also shown to enhance anti-PD1 treatment in the sarcoma and colorectal carcinoma models (MCA1956 and MC38). Both models exhibited partial tumor rejection under anti-PD1 treatment [38]. However, the combination of anti-PD1 and TREM2-deficiency or TREM2 blockade with the 178 mAb led to a 100% tumor rejection. This indicates that TREM2 inhibition significantly amplifies the efficacy of anti-PD1 immunotherapy. 

Of note, the antibody treatment used in our study did not induce macrophage depletion but rather skewed the macrophage phenotype within the TME. Although the 178 mAb showed an ability to inhibit TREM2 function *in vitro* by blocking the ligand binding [38], the exact mechanism of action *in vivo* needs to be fully elucidated. A different group successfully depleted TREM2^+^ macrophage using an effector-enhanced anti-TREM2 antibody (eFc mAb) [74]. In the EMT6 breast cancer and the ID8 ovarian cancer model, eFc mAb treatment significantly reduced tumor growth and a number of TREM2^+^ macrophages [74]. In the anti-PD-1 resistant colon carcinoma model CT26, the combination of anti-TREM2 and anti-PD-1 elicited tumor rejection in 20–60% of treated mice, along with increased infiltration of activated CD8 T cells [74]. Residual TAMs also exhibited a more inflammatory phenotype, with an increased expression of co-stimulatory molecules [74]. The translational potential of TREM2 targeting using a depleting antibody is being further assessed in human settings with an ongoing clinical trial testing the effect of the anti-TREM2 depleting mAb PY314, as a single agent or in combination with Pembrolizumab (NCT 04691375) [74]. 

Taken together, TREM2 is emerging as a promising target for immunotherapy treatments, especially if combined with ICT [38,78]. 

In liver tumors, divergent roles of TREM2 were reported. As discussed above, TREM2 expression was generally associated with immunosuppression and poor prognosis in different types of tumors. In liver cancer cohorts, high TREM2 expression was correlated with inferior overall and disease-specific survival, as well as disease stage [66,74]. However, TREM2-deficient mice exhibited increased lesion number and dimension in the DEN-induced HCC model [79]. TREM2 deficiency was associated with increased acute liver damage, increased inflammatory mediators, and less fibrosis, and yet increased tumor development and progression. The authors suggest that the protective role mediated by TREM2 in HCC depends on different mechanisms, including the limitation of DNA damage response and neoplastic transformation and proliferation [66]. Moreover, the same group showed that TREM2 was expressed in human and murine hepatic stellate cells (HSCs) during tissue injury and proposed that TREM2-expressing HSCs had a beneficial effect in the HCC model [66,80]. The hepatic environment might therefore represent a unique site where TREM2 impact on tumor growth is influenced by tissue-specific factors and cell types, and this still needs to be further investigated. 

There are still several open questions about TREM2 biology that remain to be addressed and will contribute to the development of effective treatment strategies. The molecular mechanism by which TREM2 impacts TAM phenotype and function is still unknown, as well as TREM2 ligands within the TME. Next, the origin of TREM2^+^ TAMs still needs to be formally clarified with fate-mapping studies. Moreover, the potential impact of soluble TREM2 in the context of cancer needs to be further elucidated.

## 4. Future Perspectives

TREM2 targeting represents a promising approach for re-establishing protective anti-tumor immunity and overcoming resistance to current therapies. So far, the effect of TREM2-macrophage elimination is under investigation in patients with solid tumors, using a depleting mAb (PY314) alone or in combination with anti-PD1 (NCT 04691375) [74]. 

Further studies are needed to elucidate several aspects of anti-TREM2 treatment. First, the approach utilized to target TREM2 might be further explored. Strategies to reprogram macrophages to regain an anti-tumor effect might be more beneficial than macrophage elimination alone in some cohorts. Second, different treatment strategies will reveal the benefit of targeting TREM2 in combination with standard therapies (chemotherapy, radiation therapy, etc.), targeted therapies, or immunotherapy treatments. Given the heterogeneity of TME and the abundance of immunosuppressive mechanisms, combinational immunotherapy strategies targeting both myeloid and lymphoid compartments hold great potential. In this regard, the possibility of a redundant effect of anti-TREM2 and therapies that target other myeloid receptors should be taken into consideration. Third, ongoing and future analyses will help to understand how the efficacy of TREM2 modulation could be affected by different stratification parameters, including disease type or subtype, stage, TREM2 expression level, and/or TREM2^+^ macrophage localization. In fact, the development of methods to stratify patients and predict the specific response to various treatments and combination treatments represents a crucial step in exploiting the potential of promising approaches that have greatly improved the oncological field but still have partial efficacy. Finally, multiple studies highlighted that TREM2 expression in treatment-naïve samples was associated with worse survival and poor response to therapy, suggesting that TREM2 expression could be used as a possible prognostic marker.

## Data Availability

Not applicable.
